# Interaction of Calmodulin with TRPM: An Initiator of Channel Modulation

**DOI:** 10.3390/ijms242015162

**Published:** 2023-10-13

**Authors:** Kristyna Vydra Bousova, Monika Zouharova, Katerina Jiraskova, Veronika Vetyskova

**Affiliations:** Institute of Organic Chemistry and Biochemistry of the Czech Academy of Sciences, Flemingovo Namesti 2, 16000 Prague, Czech Republic; monika.vargova@uochb.cas.cz (M.Z.); katerina.jiraskova@uochb.cas.cz (K.J.); veronika.vetyskova@uochb.cas.cz (V.V.)

**Keywords:** TRPM channels, calmodulin, calcium homeostasis, calmodulin binding site, regulation

## Abstract

Transient receptor potential melastatin (TRPM) channels, a subfamily of the TRP superfamily, constitute a diverse group of ion channels involved in mediating crucial cellular processes like calcium homeostasis. These channels exhibit complex regulation, and one of the key regulatory mechanisms involves their interaction with calmodulin (CaM), a cytosol ubiquitous calcium-binding protein. The association between TRPM channels and CaM relies on the presence of specific CaM-binding domains in the channel structure. Upon CaM binding, the channel undergoes direct and/or allosteric structural changes and triggers down- or up-stream signaling pathways. According to current knowledge, ion channel members TRPM2, TRPM3, TRPM4, and TRPM6 are directly modulated by CaM, resulting in their activation or inhibition. This review specifically focuses on the interplay between TRPM channels and CaM and summarizes the current known effects of CaM interactions and modulations on TRPM channels in cellular physiology.

## 1. Introduction

The human genome encodes hundreds of integral proteins in plasma membranes called ion channels that allow rapid permeation of ions across the membrane in a highly regulated manner [1,2]. Their transport pores open and close in response to the influence or direct binding of extrinsic or intrinsic agonists and antagonists [3]. The specific structure and functional properties differ substantially. Therefore, ion channels are divided into channel families and subfamilies according to their function or sequence homology. In this review, we focus on the transient receptor potential (TRP) channel family, specifically the melastatin TRP channels (TRPM) subfamily, which plays a key role as a versatile sensor enabling individual cells and whole organisms to detect and interpret various environmental stimuli [3,4]. TRP channels play an important role in processes that often generate the intracellular Ca^2+^ signal [5]. Downstream Ca^2+^ sensor proteins monitor changes in free intracellular Ca^2+^; after ions bind a signaling molecule, the protein induces conformational changes that modulate a number of downstream signaling pathways. Calmodulin (CaM) is one of the key protagonists of evolutionarily conserved Ca^2+^ sensors. Commonly, CaM binding to TRP channels can modulate their activity in several ways. In some cases, CaM binding to TRP channels can inhibit their activity. This inhibition may involve direct physical interactions that block the channel’s pore or reduce its calcium (Ca^2+^) permeability. In other cases, CaM binding can facilitate TRP channel activity. This facilitation might involve changes in the channel’s gating properties, such as its open probability or sensitivity to other regulatory factors. CaM can sensitize TRP channels to other modulators or stimuli. This means that the presence of CaM can make TRP channels more responsive to changes in Ca^2+^ levels or other signaling molecules [1,6,7]. In addition to a brief overview of the TRPM subfamily, we focus on the interactions between TRPM channels and one of their important intracellular modulators—CaM—together with the direct and/or indirect effects of CaM on TRPM channel’s activation and inhibition.

## 2. TRP Channels

Within the intricate realm of cellular biology lies a captivating group of proteins known as TRP channels. These mysterious gatekeepers play a pivotal role in mediating the sensations that underpin our interactions with the environment. First discovered in the early 1990s, TRP channels have since emerged as essential players in a wide range of physiological processes, ranging from temperature sensing and pain perception to vision and taste [4]. The captivating journey into the world of TRP channels begins with their diverse and ubiquitous presence across various organisms, from simple unicellular organisms to complex multicellular organisms such as humans. Their evolutionary conservation underlines their importance and highlights their significant contribution to fundamental life processes.

The archetype of TRP channels was discovered in *Drosophila melanogaster*, where photoreceptors carrying TRP gene mutations showed altered vision in the presence of constant bright light [3,4,8]. Members of the TRP superfamily are found in yeast and other multicellular organisms (invertebrates and vertebrates) and are widely expressed in different cell types and tissues with varying expression in plasma and intracellular membranes. TRPs are involved in a wide range of physiological processes, such as hypertension, visceral nociception, and cytokine production [2]. In humans, TRP channels play a key role in smell, taste (bitter, sweet, and umami), vision, touch, and our ability to detect heat, warmth, and cold [8]. Mutations in several TRP genes have been implicated in various diseases such as cardiovascular disease, neurodegenerative disorders, skeletal dysplasia, renal disorders, asthma, pain, cancer, dermatological conditions, and metabolic disorders (obesity and diabetes) [6,9,10]. Twenty-eight TRP channels have been described in mammals and are divided into six subfamilies based on the varying degrees of their sequence homology (the sequence identity of all TRP channels is only 20%): TRPC (canonical), TRPV (vanilloid), TRPA (ankyrin), TRPM (melastatin), TRPML (mucolipin) and TRPP (polycystic) [7,8,11].

Structurally, TRP channels have a similar organization to voltage-gated ion channels (VGICs) due to their subunit organization and membrane topology. The subunits anchor two modules that are embedded in the lipid bilayer. The first module, the voltage sensor-like domain (VSLD), is composed of four helices (S1–S4), and the second module, the pore domain, is composed of two helices (S5–S6) and an intermediate loop. The helices formed by S4 and S5 are connected via a linker that consists of a short amphipathic helix. The major fourfold symmetry occurring in TRP is presented around the central ion permeation pathway. It is made up of pore modules that are formed by four subunits. These subunits, composed of transmembrane helices S1–S6, can form homo-tetramers as well as hetero-tetramers [7,12]. TRP channels, as membrane proteins, have contributed to the revolution in structure determination by cryo-EM. TRPV1 was the first integral membrane protein whose structure was determined using cryo-EM [12].

TRPs are noted as polymodal channels that can be activated by several distinct physical stimuli and chemical ligands [3]. TRPs mostly mediate the influx of monovalent or divalent cations but a majority of TRPs are non-selective Ca^2+^-permeable cation channels [13,14] and thus form a superfamily of channels contributing to the regulation of Ca^2+^ homeostasis [15]. Activated TRP channels alter the membrane potential, leading to a change in intracellular Ca^2+^ concentration, which plays a central role in many fundamental cellular pathological processes including muscle contraction, transmitter release, cell proliferation, gene transcription, and cell death [16]. Mutations in TRP channel genes interfere with normal patterns of Ca^2+^ distribution and are associated with hallmarks of cancer pathophysiologies [13].

## 3. TRPM Subfamily

**Figure 1:**
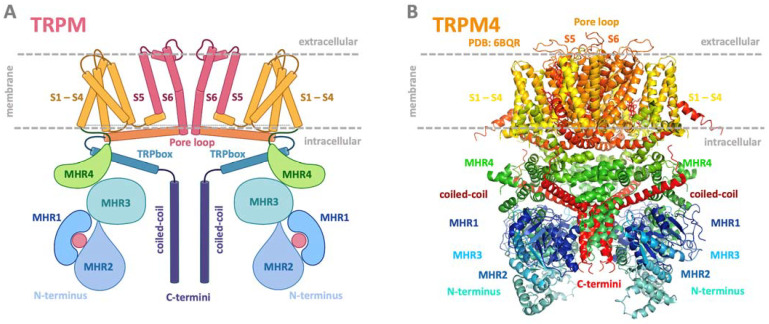
Common schematic and representative structure of the TRPM channel. (**A**) Common membrane topology of TRPM in a dimer visualization. The TRPM monomeric unit consists of six transmembrane helices (orange and pink helices), with a pore region between helices five and six (channel centre, pink part). The pore serves to transport monovalent and divalent ions. The intracellular N- and C-termini modulatory domains present in blue and green bulbs are key players in binding intracellular regulatory molecules and changing the structural conformation of the entire channel to open or close the pore region for ion transport. The pink ball in the TRPM scheme at the N-termini location represents a potential ligand binding site. (**B**) Side view of the structure of TRPM4 structure (PDB: 6BQR). Both representations show the transmembrane part of the TRPM/TRPM4 channel in yellow/orange/red; N-termini in green/blue; and C-termini intracellular domains in deep violet and red. (**A**) was adapted from the “TRPM 2 channel” template by BioRender.com, retrieved from “https://app.biorender.com/biorender-templates (accessed on 20 July 2023); (**B**) was generated using PyMol software, version 1.20 [38].

The TRPM subfamily is the largest, and its eight members (TRPM1-TRPM8) have diverse physiological functions and biophysical properties [17]. Its members are widely expressed in various cells and tissues, such as sensory ganglia, pancreatic beta cells, immune cells, tongue, heart, and kidney, and are crucial to sensory physiology [6]. Based on amino acid (AA) sequence similarity, the channels have been classified into several subgroups: TRPM1/3, TRPM4/5, TRPM6/7, TRPM2, and TRPM8. TRPM2 and TRPM8 are not located in any subgroup, although they are most closely related [7]. In recent years, cryo-EM structures have been solved for several TRPM members (TRPM2, TRPM4, TRPM5, TRPM7, and TRPM8) in closed or partially opened conformations [18,19,20,21,22,23,24,25,26]. Like all other TRP channels, they consist of four monomers composed of six transmembrane domains, four N-terminal homology regions (MHR 1–4), and a C-terminal TRP box and coiled-coil domains (Figure 1) [3]. TRPM2, TRPM6, and TRPM7 have enzymatic domains at the C-termini and have been named “chanzymes” [27,28,29].

The TRPM subfamily is associated with various human pathophysiological processes leading to organ dysfunction, cancer development, and neurodegenerative or cardiovascular diseases [2]. TRPM channels have been recognized as promising therapeutic targets, and understanding their regulation represents a fundamental building block in the development of potential therapeutics. Significant modulatory activity of TRPM channels in response to changing intracellular or extracellular Ca^2+^ levels has been reported to be controlled by a ubiquitous Ca^2+^ binding CaM [30,31,32,33,34,35]. Several members of the TRPM channel subfamily have been identified as CaM-regulated [32,33,34,36]. CaM-mediated regulation of these channels plays critical roles in various physiological processes, such as Ca^2+^ homeostasis, neurotransmitter release, and sensory perception [6]. Moreover, interactions between TRPM channels and CaM have been linked to numerous pathological conditions, including cardiac arrhythmias and neurodegenerative diseases [2,37].

CaM regulatory and signaling pathways in cells are commonly understood as key checkpoints for cellular Ca^2+^ homeostasis. TRPM channels contain multiple CaM binding domains at the intracellular N- and C-termini of the channels with an activating or inhibitory effect. A deeper understanding of TRPM regulation by CaM would require modulatory characterization of the appropriate CaM binding sites and more structural analyzes of TRPM-CaM complexes. In this review, we summarize current knowledge on discovered TRPM members and CaM interactions, with described direct and indirect effects of CaM on TRPM channel activity. As the modulation of TRP channels has gained attention in recent years [39], this review can serve as an overview of the current knowledge of the CaM/TRPM relationship to help advance the information into the application area of potential TRPM drug discovery, given that the channels are associated with many serious diseases [2].

## 4. Calmodulin

The complex organization of basic cellular processes relies on the strict regulation of intracellular Ca^2+^ levels. Basal cytoplasmic Ca^2+^ concentration is maintained at 100 nM and increases up to 1–10 μM upon induction of Ca^2+^ influx from cellular compartments or extracellular fluids [40]. This two-lobe globular protein provides hundreds of intracellular regulatory pathways, including connection with TRP channels. CaM is involved in the control of cell growth, proliferation, motility, apoptosis, etc. [41]. Human CaM is encoded by three independent genes (CALM 1-3) [42] where only a single mutation might cause life-threatening cardiac arrhythmia syndromes called calmodulinopathies [43].

**Figure 2:**
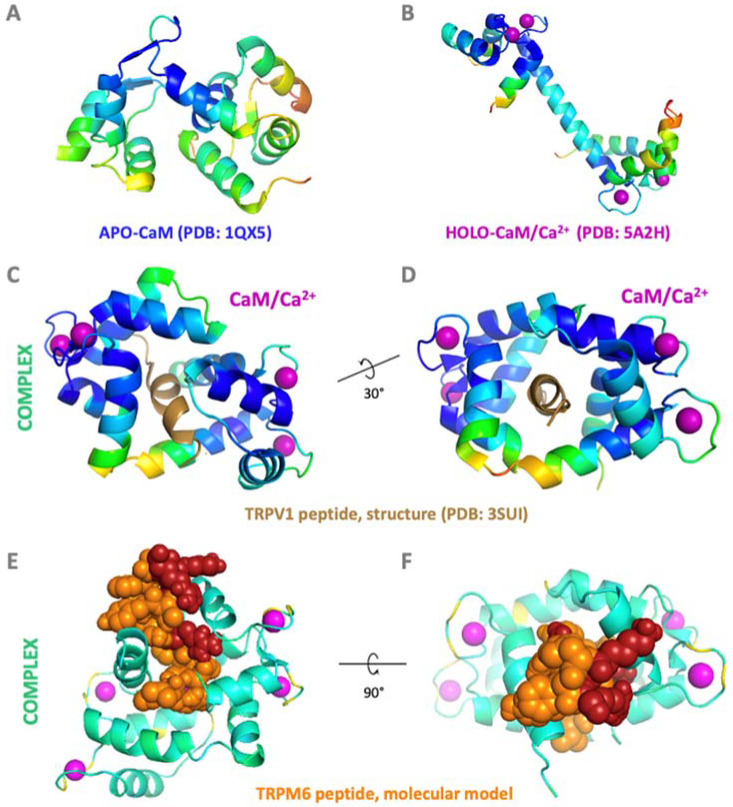
CaM complex formations with TRP channel binding sites. (**A**) Structure of apo-CaM (PDB: 1QX5) and (**B**) holo-CaM complexed with Ca^2+^ (PDB: 5A2A). (**C**) Side and (**D**) front view of the interface of the CaM/Ca^2+^—TRPV1 peptide (PDB: 3SUI) complex in the backbone representation. (**E**) Side and (**F**) front view of the CaM/Ca^2+^—TRPM6 peptide complex interface in sphere representation as a result of molecular modelling and molecular dynamics simulations (MDs) [72]. TRPM6 binding site in orange (sphere representation; red represents basic AA residues; LIGRAY**R**SNYT**RK**HF**R** (bold) confirmed to be involved in the salt bridge formations with their CaM-binding counterparts). Color convention: CaM backbone shown in rainbow colors according to *CA atoms; pink spheres represent Ca^2+^; TRPV1 peptide backbone is shown in brown; TRPM6 peptide in ball orange/red representation.

The Ca^2+^ free form, called apo-CaM, consists of two homologous globular domains (N- and C-termini lobes) connected by a central flexible linker (Figure 2A) [44]. Each CaM lobe senses changes in Ca^2+^ concentration by a pair of Ca^2+^-binding motifs called EF hands, leading to a 1:4 stoichiometry of CaM/Ca^2+^ interaction (Figure 2B) [45]. Canonical EF hands fold into a helix–loop–helix structure upon CaM–Ca^2+^ complex formation, where an acidic loop of 12 AAs provides oxygen atoms to coordinate Ca^2+^ [46]. Binding of Ca^2+^ to the EF loop changes the interhelical angle between the α-helices of the EF hand and initiates a closed-to-open transition in both lobes of CaM, associated with the exposure of hydrophobic patches. Such hydrophobic regions of the CaM–Ca^2+^ complex often facilitate interactions with downstream protein targets [47]. The CaM molecule exhibits a considerable degree of conformational plasticity, giving rise to a myriad of binding modes from compact (Figure 2C–F) [48,49] to extended ones [50,51].

CaM modulates the activity of TRP channels through activation [34,52] or inhibition [53,54,55,56,57] of signaling pathways. It can also exhibit a dual mode of action towards TRP channels, switching from an activating channel to an inactivating one upon changing Ca^2+^ concentration [58]. CaM also mediates Ca^2+^-dependent modulation of TRP channels indirectly via Ca^2+^-CaM-dependent protein kinase II (CaMKII) [59,60,61]. CaMKII participates not only in signaling cascades upstream from TRP channels, but also promotes regulation of countless downstream signaling pathways involved in the control of key cellular functions such as autophagy, cardiomyocyte contraction, or maturation of growth plate chondrocytes during bone development [37,62,63]. Apo-CaM typically does not have significant regulatory effects on TRP channels. To regulate TRP channels or other target proteins, CaM typically needs to bind Ca^2+^, and it is the CaM–Ca^2+^ complex that plays a key role in mediating the regulatory effects. In the absence of Ca^2+^ binding, apo-CaM is less likely to have a significant impact on TRP channel activity [64].

CaM recognition sites in target proteins lack a well-defined CaM-binding AA consensus. CaM binding motifs might be predicted based on shared biophysical and biochemical properties, such as the presence of a minimum of two hydrophobic AA residues, a net-positive-charged AA, and α-helical propensity [64,65]. However, these predispositions are not always mandatory for CaM–TRPM complex formation. The interaction between CaM and downstream signaling molecules usually depends on a Ca^2+^-activated CaM. These specific Ca^2+^-dependent CaM-binding motifs are classified, according to the distance between the hydrophobic residues, into two major classes: hydrophobic positions 1-10, 1-14, and 1-5-8-14 [65,66,67]; and four minor classes: 1-16 [68], 1-17 [69], 1-18 [70] and short 1-3 [71] motifs. Most CaM/target complexes are Ca^2+^-dependent; however, several proteins might also interact with apo-CaM by the so-called IQ motif defined by the consensus AA sequence IQXXXRGXXXR [64]. Based on these characteristics, numerous CaM binding sites have been identified through the TRP family, e.g., the TRPM6/CaM/Ca^2+^ complex as demonstrated in (Figure 2E,F). In the following chapter, we have described these binding sites specifically for TRPM members.

## 5. CaM Binding Domains at TRPM Channels

Interactions of TRP channels with CaM are profoundly maintained by TRP-present clusters of hydrophobic and positively charged AA residues. Alanine scanning mutagenesis of basic residues in the TRP binding regions disrupted complex formation, as reported in many studies of CaM complexes [73,74,75,76]. Hydrophobic as well as positively charged AA residues are often located at TRP binding regions with specific positions characterized as binding motifs [65]. CaM binding sites are commonly predicted based on a hydrophobic binding motif located on the interacting protein partner, which characterizes the accessible binding region to hydrophobic counterparts from CaM.

In this review, we list all TRPM binding sites for CaM that have been characterized in vitro. Table 1 shows the AA sequences of TRPM binding regions with the respective dissociation constants discovered for CaM complexes using biophysical methods [25,36,37,39,40]. Briefly, an identical hydrophobic binding motif was found for TRPM4np1 and TRPM5np (TRPM4 channel, UniProtKB/SwissProt: Q8TD43, V129-Q147). The 1-5-10 hydrophobic motif is localized at positions L134-L138-V143 (TRPM4np1) and L84-L88-V93 (TRPM5np). The highest sequence similarity of hydrophobic AA residues was identified for TRPM6np and TRPM7np, where the binding regions share an identical 1-5-10 hydrophobic binding motif at positions Y525–Y529–F534 (TRPM6np) and Y524–Y528–F533 (TRPM7np).

**Figure 3:**
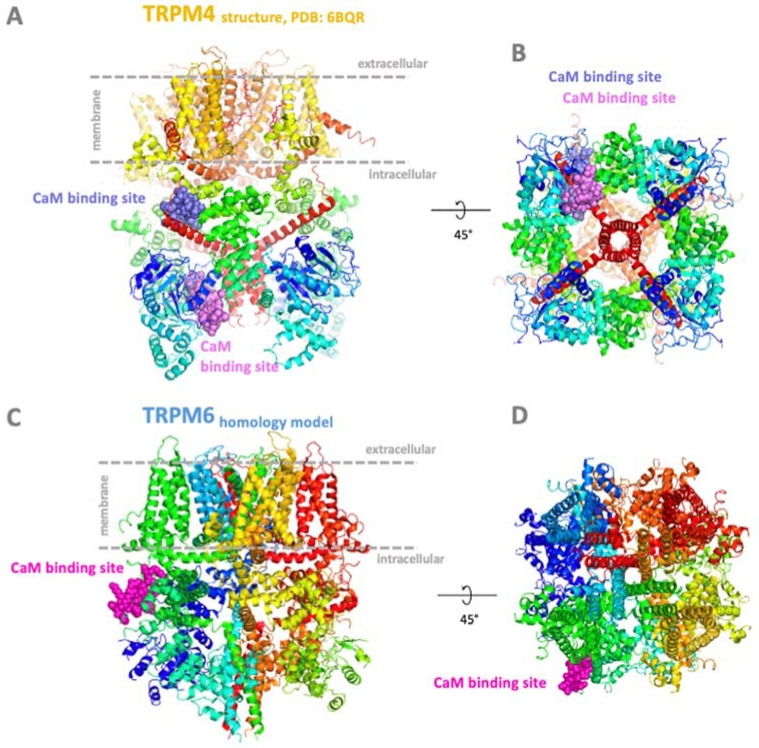
Localization of representative CaM binding sites at the TRPM4 and TRPM6 N-termini intracellular tails. (**A**,**C**) Side and (**B**,**D**) bottom view of TRPM4 structure ((**A**,**B**) PDB: 6BQR) and TRPM6 homology model (**C**,**D**) [22,72] with identified highlighted CaM binding sites accessible to CaM from intracellular cell environment [74,75]. Color convention: TRPM4 and TRPM6 backbones shown in rainbow ribbon according to *CA atoms; CaM binding sites of TRPM channels shown in pink and violet ball representations.

The hydrophobic TRP binding motifs for CaM also mostly contain positively charged AA residues that play a key role in the interactions with CaM binding partners [51,69,75]. These clusters present at TRP channels commonly bear specific patterns in their positions [73,74,78,79]. Multiple sequence alignment of TRPM binding regions [77] revealed consensus sequences of basic AA residues (RxxxxR/K, where x is any AA) confirming key players for CaM complex formations. For example, the alignment revealed a strong consensus of R and K in all TRPMs’ basic residues at CaM binding regions. TRPM5np K90 shows the character of basic residue identity with TRPM4np1 and TRPM1np. The TRPM5np binding region contains hydrophobic AAs that form two possible CaM-binding motifs W83-V87-L92 [1-5-10 motif] and L84-L88-V93 [1-5-10 motif] with four embedded basic AAs (R85, R89, K90, and K94). The exception is the TRPM5np R85, which does not fit into consensus with any TRPMs’ binding epitopes. TRPM7np and TRPM6np contain four basic residues, R525-R530-K531-R534 in TRPM7 and R526-R531-K532-R535 in TRPM6, at the same position with respect to the hydrophobic motif. Sequence alignment confirmed a similar pattern of position of hydrophobic and basic AA residues, suggesting a CaM binding motif for such regions across all TRPM members. The position of these CaM binding motifs is often placed at the surface or in the pore place of TRPs (Figure 3).

## 6. CaM Binding Domains of TRPM Channels Associated with Diseases

Specific mutations in the CaM binding domains of TRPMs have been associated with various diseases and disorders. These mutations led to dysregulated channel activity, disrupted Ca^2+^ signaling, and, consequently, contributed to pathophysiological conditions. Mutations in CaM binding domains of TRPM channels have been linked to cardiac conduction disorders (TRPM4), including familial progressive cardiac conduction defects [80]. These mutations can disrupt normal Ca^2+^-dependent regulation of TRPM4 and affect the electrical conduction system of the heart. Moreover, function-related TRPM4 mutations (I1033M, I1040T) were also linked to erythrokeratodermia [81]. Mutations in TRPM6, which plays a role in magnesium and Ca^2+^ homeostasis, can lead to hypomagnesemia with secondary hypocalcemia. These mutations may interfere with the binding of CaM to TRPM6, affecting its function in regulating magnesium and Ca^2+^ absorption in the intestine and kidney [82,83].

Mutations in the CaM binding domain of other TRP members were also associated with serious pathological conditions. The TRPV4 channel have been linked to skeletal dysplasia, where such mutations can result in abnormal Ca^2+^ signaling, affecting skeletal growth and development [84]. Mutations in the CaM binding domain of TRPC6 have been associated with focal segmental glomerulosclerosis, a kidney disorder characterized by scarring of the glomeruli. These mutations resulted in increased TRPC6 activity, which contributes to abnormal Ca^2+^ influx and cellular damage in kidney cells [85].

These examples illustrate how mutations in the CaM binding domains of TRP channels can lead to various diseases or disorders by disrupting the normal regulatory mechanisms of these channels. Dysregulation of Ca^2+^ signaling, which is often mediated by CaM, can have widespread effects on cellular processes and physiological functions, contributing to disease pathogenesis. It is important to note that ongoing research continues to uncover the roles of these mutations in disease development and progression.

## 7. First Structural Analysis of CaM in Complex with TRP

The explosion of cryo-electron microscopy (cryo-EM) techniques has enabled the solving of many previously unattainable channel classes including TRPMs [18,20,21,22,23,24,25,26]. This technique also helped to decode the first structures of TRP channels with CaM [86,87]. Specifically, the structure of CaM with TRPV6 and TRPV5 revealed an unexpectedly intricate binding interface where CaM is buried into the TRPV pore space. CaM plays a crucial role in modulation of the activities of TRPV and TRPC channels by binding to their C-terminal tail domains. Since the structure of the TRPM–CaM complex has not been solved yet, this chapter delves into the structural intricacies of the first solved complexes of CaM and the three TRP channels—TRPV5 [86,87], TRPV6 [88], and TRPC4 [89].

**Figure 4:**
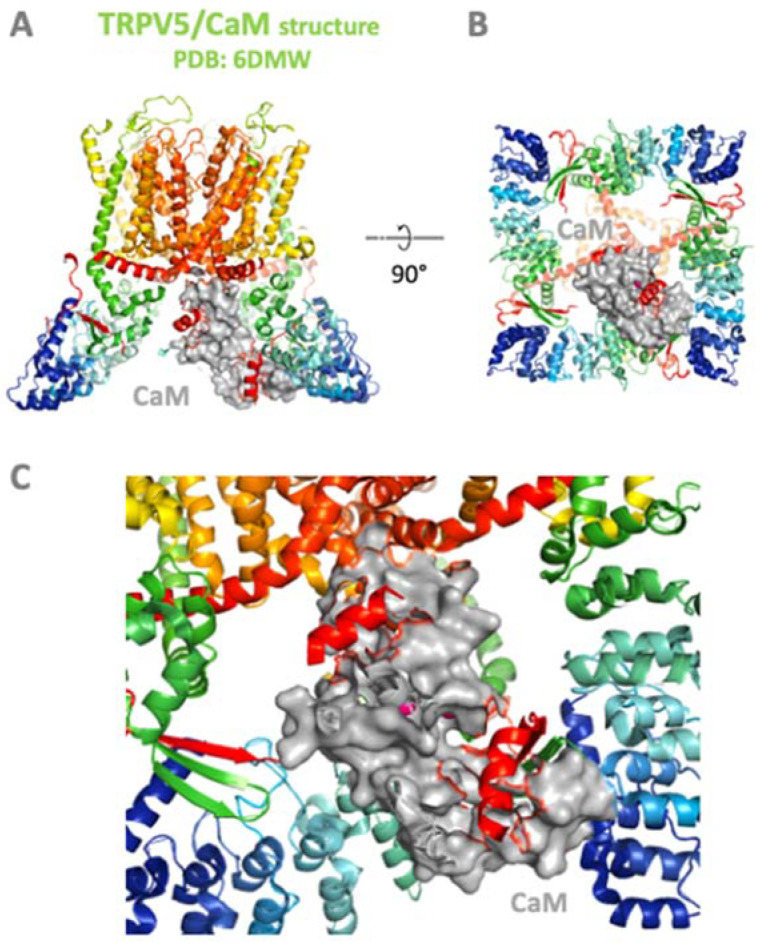
Structure of TRPV5 in the complex with CaM-Ca^2+^ (PDB: 6DMW) [87]. Cartoon representation of (**A**) side and (**B**) bottom views of CaM-bound TRPV5. (**C**) A detailed view of CaM bound in the complex with TRPV5 revealed two binding sites for N- and C-lobes of the CaM. Color convention: TRPV5 backbone in rainbow ribbon spectrum according to C-alpha; CaM in a surface representation, grey color; Ca^2+^ in the complex with CaM are shown as a magenta ball representation.

Recent structural studies using cryo-electron microscopy (cryo-EM) and X-ray crystallography have provided valuable insights into the interactions between CaM and the C-termini tails of TRPV5 and TRPV6 channels [86,87]. The analyses revealed CaM’s binding mode to a specific amphipathic helix in the C-terminal domain of the channels, with the CaM molecule engaging two binding sites within. The TRPV5 (Figure 4) and TRPV6 C-termini domains interact with the N- and C-lobes of CaM, respectively. Ca^2+^ binding to CaM induces conformational changes that enable the N- and C-lobes to clamp around the helical region of the TRPV C-terminal domain. This interaction not only stabilizes the channels in the closed state (channel inhibition), but also modulates their gating properties in response to Ca^2+^ signals. Structural studies also revealed that binding of CaM induces some degree of dimerization in TRPV5 and TRPV6 channels, further emphasizing the significance of these interactions in channel regulation.

Structural studies of the TRPC4–CaM complex are comparatively limited, but have revealed fundamental insights into the complex formation [89]. Cryo-EM studies indicate that in the structure of the TRPC4–CaM complex, the central core of the cytoplasmic region is occupied by a coiled-coil helix. Thus, CaM cannot access the core of the cytoplasmic region in TRPC4. Binding of CaM to TRPC4 regulates the gating properties of the channel (inhibition upon CaM interaction), thereby influencing Ca^2+^ influx and downstream signaling pathways.

## 8. CaM Modulates the Activity of TRPM Members

### 8.1. TRPM1 and TRPM3

The first identified mammalian TRPM, TRPM1, was originally named melastatin because its expression levels were inversely correlated with metastatic potential in some melanoma cell lines [90]. Human TRPM1 mutations are associated with congenital stationary night blindness, when patients lack rod function and suffer from night blindness from early childhood. In addition to the function of transduction cation channels, TRPM1 is one of the retinal autoantigens in some paraneoplastic retinopathies associated with retinal ON-bipolar cell dysfunction. The TRPM1 channel in terms of potential regulatory function was recently associated with AKT activation, colony formation, cell mobility, and xenograft tumor growth in melanoma cells. TRPM1 elevated cytosolic Ca^2+^ levels and activated CaMKIIδ (Ca^2+^/calmodulin-dependent protein kinase IIδ) to promote CaMKIIδ/AKT interaction and AKT activation [61]. The direct binding of CaM to TRPM1 has not been described yet. The only direct Ca^2+^-dependent interaction of TRPM1 was described with S100A1, although the specific modulatory function is not known yet [79].

TRPM3 is a Ca^2+^-permeable non-selective cation channel with a pivotal role in the detection of noxious heat in dorsal root and trigeminal ganglia [91]. TRPM3 channels participate in blood vessel contraction and smooth muscle proliferation [92], insulin secretion by pancreatic β-cells, and renal Ca^2+^ homeostasis [93]. Substitutions in TRPM3 have been linked to intellectual disability and epilepsy [94]. The most potent agonist of TRPM3 channels is the endogenous neurosteroid pregnenolone sulphate [95]. TRPM3 activity is also regulated by the plasma membrane levels of phosphatidylinositol phosphates (PIPs), which directly potentiate TRPM3 currents (Table 2) [96,97]. Activating stimuli lead to Ca^2+^ influx into the cytoplasm, and elevated Ca^2+^ levels provide a negative feedback signal to TRPM3 channels.

CaM modulation of TRPM3 has not yet been described. In vitro binding assays revealed two Ca^2+^-dependent CaM-binding regions at the TRPM3 N-terminus, overlapping with interaction regions for phosphatidyl inositol 4,5-bisphosphate (PIP2) [73,98]. Recently, five separated CaM binding sites (CaMBS 1-5) at the TRPM3 N-terminus were predicted and confirmed by pull-down and dot blot analyses [33]. The CaM-binding affinities of CaMBS 1-5 increase in the presence of Ca^2+^. Interestingly, CaMBS 1 and CaMBS 3 regions overlap with two CaM-binding regions previously described by Holakovska et al. [73]. The existence of multiple CaM-binding sites with different binding affinities points to cooperativity in the complicated multi-level modulation of TRPM3 channels. In addition, the number of CaM-binding sites varies between TRPM3 isoforms, with CaMBS 2 and 3 occurring in all TRPM3 isoforms while CaMBS 1, 4 and 5 are subject to alternative splicing [33,99]. Variation in the CaM-binding sites employed by particular TRPM3 isoforms could serve as a fine-tuning tool to generate an isoform/tissue-specific response to changes in Ca^2+^ levels. However, the functional role of the identified CaM-binding domains in Ca^2+^-dependent regulation of TRPM3 has not yet been demonstrated. Patch clamp experiments indicated the involvement of CaMBS 2 in TRPM3 stabilization rather than in c-dependent regulation [33]. A recent study described CaM presence as important in the activation of TRPM3- and TRPM8-induced intracellular signaling, most likely for a direct interaction with the channels [100]. Ca^2+^ influx through TRPM3 and TRPM8 responds to TRPM3- and TRPM8-induced signaling by activating the calmodulin-regulated enzyme calcineurin, which acts as a negative feedback loop for TRPM3 and TRPM8 channel signaling.

**Table 2:** Overview of TRPM members modulated by CaM.

TRPM Member	CaM Modulation	Activation/ Inhibition by CaM	Modulation by Other Agents	Reference
TRPM1	NO	-	-	-
TRPM2	YES	activation	Ca^2+^	Tong et al., 2006 [34]
TRPM3	NO	inhibition	PIP2, calcineurin	Toth B. et al., 2015 [96]
TRPM4	YES	activation, inhibition	ATP, PKC, Ca^2+^	Nilius B. et al., 2005 [32]
TRPM5	NO	-	Ca^2+^, PIP2	Prawitt et al., 2003; Liu et al., 2003; Zhang et al., 2007 [101,102,103]
TRPM6	NO	-	Mg^2+^, PIP2	Voet et al., 2004; Xie et al., 2011; Groenestege, W. M. et al., 2006 [104,105,106]
TRPM7	NO	-	Mg^2+^/Mg-ATP, PIP2, CaMKII	Mishra R. et al., 2009; Turlova E. et al., 2021; Runnels L.W. et al., 2002; Nadler R. et al., 2001 [31,35,107,108]
TRPM8	YES	activation; inhibition	Ca^2+^, PIP2, PIRT, calcineurin	Diver M. et al., 2019; Sisco N.J. et al., 2020 [20,109]

### 8.2. TRPM2

The TRPM2 channel is a non-selective, Ca^2+^-permeable cation channel, widely expressed in the CNS [110] but also in heart and endothelial cells [111,112], pancreatic β-cells [113], and immune cells [114]. TRPM2 functions as a potent cellular oxidative stress sensor activated by the second messenger adenosine diphosphoribose (ADPR), which is generated in mitochondria in response to oxidative stress [115]. TRPM2 sensitivity to ADPR is largely facilitated by Ca^2+^. Intracellular Ca^2+^ acts as a crucial modulator of ADPR-mediated TRPM2 gating and provides a positive influence on TRPM2 [116]. Whole-cell patch clamp experiments revealed the participation of CaM in Ca^2+^-dependent ADPR-induced TRPM2 currents [117]. CaM/Ca^2+^ associates with the IQ-like motif occurring in the TRPM2 N-terminus and alanine-scanning mutagenesis of the key residues (Table 2) [34]. Mutations of the IQ-like motif in the context of the full-length TRPM2 resulted in decreased association with CaM/Ca^2+^, linked with reduced Ca^2+^ currents and a confirmed role of the IQ-like motif in TRPM2 sensitization.

In addition to sensing oxidative stress, TRPM2 also functions as a thermal sensor and regulator [118,119]. Potentiation of TRPM2 by temperature change alone requires elevation above 47 °C [120]. TRPM2 currents evoked by ADPR binding to its C-terminal NUDT9 homology (NUDT9H) domain [121] have been shown to be potentiated by exposure to lower temperatures (above 35 °C) [122], pointing to a coupling of ADPR, Ca^2+^, and temperature modulation of TRPM2. A novel Ca^2+^-dependent CaM binding site localized in the NUDT9H domain of TRPM2 binds CaM/Ca^2+^ at 37 °C but not at room temperature [30]. Alanine scanning mutagenesis of a peptide derived from the binding site confirmed key AA residues of the CaM-binding motif and patch clamp experiments and indicated participation of the described CaM binding motif in thermal sensation through TRPM2. The proposed temperature-dependent mode of regulation suggests a partial unfolding of the NUDT9H domain at temperatures above 35 °C with subsequent CaM/Ca^2+^ binding to the exposed binding epitope [30]. This is consistent (1) with previous work in which no tight binding of CaM/Ca^2+^ to the TRPM2 C-terminus was detected at room temperature [34] and (2) with the TRPM2 cryo-EM structure, where the CaM-binding site in NUDT9H domain is probably not accessible to CaM [24]. Recently, indirect CaM modulation of TRPM2 has been described in the field of hepatocellular carcinoma (HCC) [123]. TRPM2 promotes HCC cell proliferation through activation of the Ca^2+^-CaM-CaMKII signaling pathway to induce the expression of key G1/S regulatory proteins and accelerate the cell cycle of cancer cells.

### 8.3. TRPM4 and TRPM5

The TRPM4/TRPM5 subgroup of TRPMs comprises phylogenetically related channels with a high degree of sequence similarity [124]. Despite sharing several key characteristics, their expression profiles and roles in physiological processes differ. TRPM4 represents an intensively studied channel in terms of its structure and functional regulation. It acts as a Ca^2+^-impermeable non-selective monovalent cation channel expressed in a wide variety of tissues, including brain, heart, intestine, stomach, prostate, and lung [125]. TRPM4 channels are involved in various physiological processes, such as T cell activation, myogenic vasoconstriction, allergic reactions, neurotoxicity, etc. [125,126,127]. Dysregulation of TRPM4 has been associated with life-threating conditions, such as neurodegenerative diseases [128] and colorectal, prostate, breast, and cervical cancer [129] as well as with cardiac conditions [130,131,132] such as arrhythmias, hypertrophy, and ischemia-reperfusion injuries. TRPM4 claims more and more attention due to discovered pathologies, and, together with these findings, hides untapped potential for future therapeutics. The cryo-EM structure of TRM4 (Figure 1) revealed an inverted crown-like tetrameric architecture with a transmembrane core formed by six transmembrane helices (S1 to S6) from each subunit [18,22,25]. Long TRPM4 N-termini contain four TRPM homology regions (MHR 1-4) and account for the majority of the large cytosolic part of TRPM4. The post-S6 TRP domain probably acts as a key element of the TRPM4 gating apparatus and provides interactions with Ca^2+^ or PIP2 [25].

TRPM4 activation depends on intracellular Ca^2+^ and leads to plasma membrane depolarization via Na^+^ entry and reduced Ca^2+^ influx via Ca^2+^ entry pathways. TRPM4 binds Ca^2+^ through acidic residues near and in the TRP domain and undergoes rapid desensitization to cytosolic Ca^2+^ [133,134]. PIP2, a potent modulator of TRPM4 sensitivity to Ca^2+^, re-sensitizes the TRPM4 channel, and its hydrolysis by Ca^2+^-activated phospholipase C is associated with TRPM4 desensitization to Ca^2+^ [135,136]. TRPM4-Ca^2+^ sensitivity is also modulated by ATP, phosphorylation by protein kinase C, and, especially, by Ca^2+^-activated CaM [32]. Patch clamp experiments revealed a dramatic decrease in Ca^2+^-dependent TRPM4 activation upon expression of Ca^2+^-binding sites with a defective CaM variant. At the same time, native CaM limited TRPM4 desensitization. Deletion of three TRPM4 C-terminal fragments with affinity for Ca^2+^/CaM resulted in impaired TRPM4 activation, which could be activated only at a 1 mM concentration of Ca^2+^. Therefore, CaM is assumed to enable activation of TRPM4 at physiological levels of intracellular Ca^2+^. Two CaM-binding epitopes derived from the TRPM4 N-terminus and one from the TRPM4 C-terminus have been described in more detail [74,75]. These epitopes overlap with the binding site for another Ca^2+^-sensor protein, S100A1, and the C-terminal one binds PIP2 as well. In addition, the C-terminal epitope overlaps with TRPM4 fragments involved in Ca^2+^/CaM-dependent sensitization of TRPM4. The functional role of the TRPM4 N-terminal epitopes remains to be elucidated. The downstream key enzyme CaMKII has recently been associated with arrhythmogenic changes in the stressed heart [137]. Pathological TRPM4 upregulation by excessive CaMKII activity may be a pivotal predisposing factor for life-threating cardiac disorders and represent an attractive target for therapeutic intervention.

TRPM5 is a voltage-dependent non-selective monovalent cation channel that precludes Ca^2+^ influx into the cytosol through plasma membrane depolarization [138]. Its expression is more restricted and occurs mainly in type II taste cells that detect sweet, bitter, and umami or in pancreatic β-cells [101]. Normal taste signaling through TRPM5 relies on a functional coupling with its closest homolog, TRPM4 [139]. TRPM5-mediated regulation of insulin release by pancreatic β-cells makes it an attractive target for type 2 diabetes mellitus therapy [140]. It has been also discovered that the TRPM5 inhibitor triphenylphosphine oxide significantly inhibited spontaneous metastasis to the lungs. Analysis in silico also suggested a significant correlation between high levels of TRPM5 expression and shorter survival in patients with melanoma and gastric cancer. Such findings indicate that TRPM5 may be a suitable potential therapeutic target, as its inhibition may prevent metastasis and prolong the overall survival of patients with melanoma and gastric cancer [141]. The cryo-EM structure of TRPM5 revealed substantial structural differences between TRPM5 and TRPM4, underlining diverse functional properties [26]. The cytosolic part of TRPM5 is mainly composed of MHR1/2 and MHR3/4 domains, with the MHR1/2 domain closer to the transmembrane domain, leading to a different interplay between subunits and a more compact assembly compared with the TRPM4 structure.

TRPM5 is directly activated by Ca^2+^ binding and undergoes even more pronounced desensitization than TRPM4 [101,138,142]. Similar to TRPM4, TRPM5 desensitization can be partially reversed by PIP2 [102,103]. TRPM5 contains two Ca^2+^-binding sites suggested to cooperatively gate TRPM5 channels [26]. The first Ca^2+^-binding site (CaBS1) occurs in the transmembrane domain area and seems conserved among Ca^2+^-gated TRPM channels. The second site (CaBS2) is probably unique for TRPM5 localized between its MHR1/2 and MHR2/3 domains. CaBS1 is considered to be a key element for TRPM5 activation, while CaBS2 may fine-tune the Ca^2+^-binding affinity of CaBS1 and modulate the voltage dependence of TRPM5. A recent study revealed a Ca^2+^-dependent CaM-binding site in the TRPM5 N-terminus overlapping with the binding site for another Ca^2+^-binding protein—S100A1 [76].

### 8.4. TRPM6 and TRPM7

TRPM6 and its close homolog TRPM7 act as gatekeepers of human Mg^2+^ homeostasis [104,143]. TRPM6 and TRPM7 possess the unique feature of a cation channel fused to an α-kinase domain that phosphorylates the ion channel itself as well as its downstream targets, e.g., receptor tyrosine kinases’ (RTK) downstream signaling molecules [144,145]. Potent bidirectional cross-talk between TRPM6 and the RTK signaling cascade appears to be associated with the onset of hypomagnesia during RTK-inhibitor-based cancer therapy [146]. TRPM6/TRPM7 α-kinase can also be cleaved and translocated to the nucleus to modulate gene expression [27,147]. Despite the many similarities shared by TRPM6 and TRPM7, they still modulate cellular functions differentially and their responses cannot be mutually compensated.

Deeper insight into the structure–function relationship has been described for TRPM7, which is a non-selective cation channel with high permeability to divalent cations including Mg^2+^, Ca^2+^, and Zn^2+^ [148]. TRPM7 functions as a ubiquitously expressed regulator of Mg^2+^ homeostasis, Ca^2+^ signaling, and cell proliferation/differentiation with a key role in embryonic development and organogenesis [148,149]. The cryo-EM structure of truncated TRPM7 (lacking the α-kinase domain) revealed a similar overall architecture as in other TRPM members [23]. However, the conformation of N-terminal cytosolic MHR regions differs between TRPM7 and TRPM6. In both TRPM7 and TRPM6, the C-terminal stretcher helix penetrates through the MHR regions to the TRP domain and could transmit signals from the MHR domains to the S6 gating helix. The activity of TRPM7 is inhibited by an increase in cytosolic Mg^2+^/Mg-ATP and PIP2 hydrolysis [107,108]. Negative feedback modulation of TRPM7 by Ca^2+^ is at least partially mediated by Ca^2+^/CaMKII [31,35]. The other study described high-conductance Ca^2+^-dependent K^+^-channel-induced hyperpolarization that likely enhances the driving force of TRPM7-mediated Ca^2+^ entry and seems to activate CaMKII accordingly [150]. Our recent study points to a direct TRPM7 interaction with Ca^2+^/CaM and Ca^2+^/S100A1; however the functional role of the newly identified TRPM7 N-terminal binding domain remains to be further investigated [77].

TRPM7 forms a homo-tetramer or assembles with TRPM6 into a hetero-tetrameric channel TRPM7/TRPM6. Like TRPM7, TRPM6 serves mainly as a Mg^2+^ influx channel, inactivated by PIP2 hydrolysis or an increase in cytosolic Mg^2+^ levels [104,105]. TRPM6 exhibits a restricted expression profile with high expression rates in intestine, lung, and kidney and its mutations have been associated with hypo-magnesia with secondary hypocalcemia [82,106]. Recent study suggests a primary role of native TRPM6 as a subunit of the heteromeric TRPM6/TRPM7 channel rather than forming a functional homo-tetramer [151]. Association with TRPM6 decreases TRPM7 Mg-ATP sensitivity and enables constitutive activity of TRPM6/TRPM7 in the physiological range of cytosolic Mg^2+^ concentrations. In addition to Mg^2+^, TRPM6 conducts Ca^2+^, albeit with lower efficiency. The N-terminus of TRPM6 contains overlapping binding epitopes for CaM and S100A1 [72]. However, CaM-dependent modulation of TRPM6 and the role of the identified CaM-binding epitope remains to be directly verified.

### 8.5. TRPM8

TRPM8 was molecularly identified in 2002 and designated as cold-sensitive receptor 1 (CMR1) [152,153]. TRPM8 is activated at lower temperatures as well as by refrigerants such as menthol, eucalyptol, linalool, icillin, and camphor. TRPM8 is widely expressed in mammal neurons’ dorsal root ganglia (DRG) and trigeminal ganglia (TG) [154,155] and in the oral mucosa [154]. The channel has been shown to be a major mediator of painful colds in humans [156].

The cryo-EM revealed TRPM8 homo-tetramers with transmembrane helices S1 to S4 forming the voltage-sensor-like domain (VSLD) and S5, S6, and the pore helix of the pore domain [21]. The channel activation relies on PIP2-induced allosteric changes in the VSLD cavity localized between S4 and the cytosolic TRP domain, which can in turn accommodate cooling agents such as menthol analogue WS-12 or icillin [19]. Activated TRPM8 conducts Ca^2+^ ions, triggering a negative feedback mechanism at high cytosolic concentrations. Ca^2+^-induced downregulation of TRPM8 is controlled by several signaling pathways. The cryo-EM structure of desensitized TRPM8 revealed direct Ca^2+^ coordination by S2, S3, and S2–S3 linker residues, accompanied by a constriction within the ion conduction pathway [20]. Mutagenesis of key Ca^2+^ coordinating residues confirmed the crucial role of direct Ca^2+^ binding in TRPM8 desensitization. Elevated cytosolic Ca^2+^ also activates PLC to hydrolyze PIP2, which is required to sustain the TRPM8 active state. In addition, TRPM8 downregulation has been associated with Ca^2+^-activated CaM [36]. CaM might directly bind and gate TRPM8 [157] or regulate TRPM8 activity through another modulatory protein—phosphoinositide-interacting regulator of TRP (PIRT) [109]. The CaM/PIRT complex assembled in a Ca^2+^-free environment seems to dissociate upon Ca^2+^ binding to CaM. Thus, CaM may control the availability of PIP2 for TRPM8 activation by sequestering/releasing PIRT in response to changing Ca^2+^ levels. The hypothesis of a CaM-PIRT-TRPM8 signaling pathway is consistent with the PIP2 dependency of CaM-mediated TRPM8 downregulation [36].

Other studies have shown that activation of PKC produced menthol-induced desensitization of TRPM8 as well as dephosphorylation and downregulation of the channel [63,64,65,158,159]. However, none of the nine putative PKC phosphorylation sites of TRPM8 were involved in channel modulation. The activation of PKC did not increase the phosphorylation state of the channel but rather activated the Ca^2+^**-** and calmodulin-dependent serine/threonine protein phosphatase calcineurin, indicating a dephosphorylation-induced desensitization process; therefore, it has been concluded that PKC has an indirect effect on menthol-induced TRPM8 desensitization [160].

## 9. Potential TRPM-CaM Therapeutic Avenues

TRPM channels such as TRPM8 and TRPM3 are involved in thermal and pain sensation. Dysregulation of their activity is implicated in conditions like neuropathic pain and migraine [160,161]. Modulating the interaction between CaM and TRPM channels could offer strategies for the development of new analgesics. Furthermore, TRPM4 and TRPM7 channels play roles in vascular tone regulation and cardiac rhythm [137,162]. Therefore, CaM-mediated modulation of these channels affects their contribution to physiological processes like smooth muscle contraction and cardiac myocyte activity. Targeting CaM-TRPM4/TRPM7 interactions could have implications for the management of cardiovascular disorders. TRPM channels are also often associated with neurological disorders such as epilepsy and neurodegenerative diseases. As CaM is intricately involved in TRPM channel regulation, interventions aimed at modulating this interaction may offer innovative approaches for neuroprotective strategies as well.

## 10. Conclusions and Future Perspectives

TRPM channels play pivotal roles in a wide array of physiological processes, including Ca^2+^ homeostasis, sensory perception, and cellular signaling. Among the factors that modulate the activity of TRPM channels, CaM stands out as a crucial regulator. Numerous CaM binding regions in TRP channels have been characterized; however, the regulatory or signaling functions of CaM in ion transports remains to be further studied. Modulation of TRP channels by CaM is clearly a common process for all members of TRP families, including TRPM. Regulatory and signaling pathways of CaM in cells are commonly understood as key checkpoints for Ca^2+^ cell homeostasis. TRPM channels elicit multiple CaM binding domains at the intracellular N- and C-termini of the channels with activating or inhibiting effects. A deeper understanding of TRPM regulation by CaM would require the modulatory characterization of suitable CaM binding sites and more structural analyses of TRPM–CaM complexes. We are currently beginning to understand how parts of protein machinery, such as transmembrane receptors, are regulated, and it is necessary to discover each piece of the puzzle to understand the complexity involved in these processes. In recent years, the number of publications mentioning binding sites and more intricate processes of interactions in ligands/receptor complexes has begun to rise [49]. The entire mechanism of membrane receptor modulation is expected to be much more complex than protein functions and interactions explained in the past.

Targeting the TRPM–CaM regulatory axis holds therapeutic potential for a wide range of diseases, making TRPM channels and their interaction with CaM an enticing area of research. The dynamic interplay between TRPM channels and CaM represents a fascinating and complex regulatory mechanism that fine-tunes cellular responses to varying Ca^2+^ levels. Elucidating the intricacies of this regulatory relationship not only advances our knowledge of cellular physiology but also paves the way for potential therapeutic interventions in diseases linked to dysregulated TRPM channel activities.

## Figures and Tables

**Table 1:** CaM binding regions of TRPM N-termini. The table shows the studied binding sites of TRPM members for CaM with confirmed formation of a specific complex. Numbers at the AA sequence indicate the location of the CaM binding site in the context of the TRPM member. The complexes were studied by in vitro testing (fluorescence anisotropy, FA; surface plasmon resonance, SPR; microscale thermophoresis, MST) with investigated K_D_ values in the range of micromolar levels; N.D. stands for Not Determined.

TRPM Member	CaM Binding Sites	Binding Affinity (KD)	Reference
TRPM2np	_399_IVEWTKKIQDIVRRRQLLTV_F419_	N.D.	Tong et al., 2006 [34]
TRPM2cp	_1352_VTHWRRNEDGAICRKSIKKMLEVL_1375_	110 (±18) nM	Gattkowski et al., 2019 [30]
TRPM3np	_193_QNFELQPKLKQVFGKGLIKAAMTTG_217_	N.D.	Przibilla et al., 2018 [33]
TRPM3np	_41_WTIRKLCHAAFLPSVRLLKAQKSWIERAFY_70_	FA: 1.29 (±0.14) μM	Holakovska et al., 2012 [73]
		SPR: 0.198 (±0.018) μM	
TRPM3np	_304_GAEVKLRRQLEKHISLQKIN_324_	FA: 0.92 (±0.17) μM	Holakovska et al., 2012 [73]
		SPR: 0.481 (±0.074) μM	
TRPM4np1	_129_VLQTWLQDLLRRGLVRAAQ_147_	FA: 1.1 (±0.2) μM	Bousova et al., 2018 [74]
TRPM4np2	_627_FGECYRSSEVRAARLLLRRCPL_648_	FA: 1.3 (±1.8) μM	Bousova et al., 2020 [75]
TRPM4cp	_1078_PFIVISHLRLLLRQLCRRPRS_1098_	FA: 2.6 (±0.5) μM	Bousova et al., 2018 [74]
TRPM5np	_83_WLRDVLRKGLVK_94_	FA, MST: 1.0 (±0.1) μM	Bousova et al., 2022 [76]
TRPM6np	_520_LIGRAYRSNYTRKHFR_535_	FA: 14.87 (±0.7) μM	Zouharova et al., 2019 [72]
TRPM7np	_526_TYRCTYTRKRFRL_535_	FA: 6.1 (±0.4) μM	Bousova et al., 2021 [77]

## Abbreviations

AA	Amino acid
ADPR	ADP ribose
Apo-	Calcium free form of CaM
Ca^2+^	Calcium
ATP	Adenosine triphosphate
CaBS	Ca^2+^-binding site
CaM	Calmodulin
CAMBS	CaM-binding sites
CaMKII	CaM-dependent protein kinase II
CaMKIIδ	Ca^2+^/calmodulin-dependent protein kinase IIδ
CMR1	Cold-sensitive receptor 1
DRG	Dorsal root ganglia
EM	Electron microscopy
FA	Fluorescence anisotropy
FFT	Fourier transform
HOLO-CaM	CaM complexed with Ca^2+^
MD	Molecular dynamics
MHR	Melastatin homology region
MST	Microscale thermophoresis
NUDT9H	NUDT9 homology domain
PBC	Periodic boundary conditions
PH	Pleckstrin homology
PIPs	phosphatidylinositol bisphosphates
PIP2	phosphatidylinositol 4,5-bisphosphate
PIRT	Phosphoinositide-interacting regulator of TRP
PME	Particle mesh Ewald
RTK	Receptor tyrosine kinases
S100A1	S100 calcium-binding protein A1
SPR	Surface Plasmon Resonance
TG	Trigeminal ganglia
TRPs	Transient receptor potential (channels)
TRPA	Ankyrin TRP subfamily
TRPC	TRP canonical
TRPV	TRP vaniloid
TRPM	Melastatin TRP subfamily
TRPML	Mucolipin TRP subfamily
TRPP	Polycystine TRP subfamily
VGICs	Voltage-gated ion channels
VSLD	Voltage sensor-like domain

## References

[1] Voets T., Talavera K., Owsianik G., Nilius B. (2005). Sensing with TRP channels. Nat. Chem. Biol..

[2] Jimenez I., Prado Y., Marchant F., Otero C., Eltit F., Cabello-Verrugio C., Cerda O., Simon F. (2020). TRPM Channels in Human Diseases. Cells.

[3] Lopez-Romero A.E., Hernandez-Araiza I., Torres-Quiroz F., Tovar Y.R.L.B., Islas L.D., Rosenbaum T. (2019). TRP ion channels: Proteins with conformational flexibility. Channels.

[4] Samanta A., Hughes T.E.T., Moiseenkova-Bell V.Y. (2018). Transient Receptor Potential (TRP) Channels. Subcell Biochem..

[5] Vangeel L., Voets T. (2019). Transient Receptor Potential Channels and Calcium Signaling. Cold Spring Harb. Perspect. Biol..

[6] Hasan R., Zhang X. (2018). Ca(2+) Regulation of TRP Ion Channels. Int. J. Mol. Sci..

[7] Zhao Y., McVeigh B.M., Moiseenkova-Bell V.Y. (2021). Structural Pharmacology of TRP Channels. J. Mol. Biol..

[8] Clapham D.E. (2003). TRP channels as cellular sensors. Nature.

[9] Nilius B., Owsianik G. (2011). The transient receptor potential family of ion channels. Genome Biol..

[10] Ciardo M.G., Ferrer-Montiel A. (2017). Lipids as central modulators of sensory TRP channels. Biochim. Biophys. Acta Biomembr..

[11] Gees M., Colsoul B., Nilius B. (2010). The role of transient receptor potential cation channels in Ca^2+^ signaling. Cold Spring Harb. Perspect. Biol..

[12] Cao E. (2020). Structural mechanisms of transient receptor potential ion channels. J. Gen. Physiol..

[13] Yang D., Kim J. (2020). Emerging role of transient receptor potential (TRP) channels in cancer progression. BMB Rep..

[14] Bertin S., Raz E. (2016). Transient Receptor Potential (TRP) channels in T cells. Semin. Immunopathol..

[15] Shapovalov G., Lehen’kyi V., Skryma R., Prevarskaya N. (2011). TRP channels in cell survival and cell death in normal and transformed cells. Cell Calcium.

[16] Pedersen S.F., Owsianik G., Nilius B. (2005). TRP channels: An overview. Cell Calcium.

[17] Huang Y., Fliegert R., Guse A.H., Lü W., Du J. (2020). A structural overview of the ion channels of the TRPM family. Cell Calcium.

[18] Winkler P.A., Huang Y., Sun W., Du J., Lu W. (2017). Electron cryo-microscopy structure of a human TRPM4 channel. Nature.

[19] Yin Y., Le S.C., Hsu A.L., Borgnia M.J., Yang H., Lee S.Y. (2019). Structural basis of cooling agent and lipid sensing by the cold-activated TRPM8 channel. Science.

[20] Diver M.M., Cheng Y., Julius D. (2019). Structural insights into TRPM8 inhibition and desensitization. Science.

[21] Yin Y., Wu M., Zubcevic L., Borschel W.F., Lander G.C., Lee S.Y. (2018). Structure of the cold- and menthol-sensing ion channel TRPM8. Science.

[22] Autzen H.E., Myasnikov A.G., Campbell M.G., Asarnow D., Julius D., Cheng Y. (2018). Structure of the human TRPM4 ion channel in a lipid nanodisc. Science.

[23] Duan J., Li Z., Li J., Hulse R.E., Santa-Cruz A., Valinsky W.C., Abiria S.A., Krapivinsky G., Zhang J., Clapham D.E. (2018). Structure of the mammalian TRPM7, a magnesium channel required during embryonic development. Proc. Natl. Acad. Sci. USA.

[24] Wang L., Fu T.M., Zhou Y., Xia S., Greka A., Wu H. (2018). Structures and gating mechanism of human TRPM2. Science.

[25] Guo J., She J., Zeng W., Chen Q., Bai X.C., Jiang Y. (2017). Structures of the calcium-activated, non-selective cation channel TRPM4. Nature.

[26] Lu W., Du J., Ruan Z., Haley E., Orozco I., Roth R., Sabat M., Myers R. (2021). Structures of TRPM5 channel elucidate mechanism of activation and inhibition. bioRxiv.

[27] Krapivinsky G., Krapivinsky L., Manasian Y., Clapham D.E. (2014). The TRPM7 chanzyme is cleaved to release a chromatin-modifying kinase. Cell.

[28] Tóth B., Iordanov I., Csanády L. (2014). Putative chanzyme activity of TRPM2 cation channel is unrelated to pore gating. Proc. Natl. Acad. Sci. USA.

[29] Montell C. (2003). Mg^2+^ homeostasis: The Mg^2+^ nificent TRPM chanzymes. Curr. Biol..

[30] Gattkowski E., Johnsen A., Bauche A., Mockl F., Kulow F., Garcia Alai M., Rutherford T.J., Fliegert R., Tidow H. (2019). Novel CaM-binding motif in its NudT9H domain contributes to temperature sensitivity of TRPM2. Biochim. Biophys. Acta Mol. Cell Res..

[31] Mishra R., Rao V., Ta R., Shobeiri N., Hill C.E. (2009). Mg^2+^- and MgATP-inhibited and Ca^2+^/calmodulin-sensitive TRPM7-like current in hepatoma and hepatocytes. Am. J. Physiol. Gastrointest. Liver Physiol..

[32] Nilius B., Prenen J., Tang J., Wang C., Owsianik G., Janssens A., Voets T., Zhu M.X. (2005). Regulation of the Ca^2+^ sensitivity of the nonselective cation channel TRPM4. J. Biol. Chem..

[33] Przibilla J., Dembla S., Rizun O., Lis A., Jung M., Oberwinkler J., Beck A., Philipp S.E. (2018). Ca(2+)-dependent regulation and binding of calmodulin to multiple sites of Transient Receptor Potential Melastatin 3 (TRPM3) ion channels. Cell Calcium.

[34] Tong Q., Zhang W., Conrad K., Mostoller K., Cheung J.Y., Peterson B.Z., Miller B.A. (2006). Regulation of the transient receptor potential channel TRPM2 by the Ca^2+^ sensor calmodulin. J. Biol. Chem..

[35] Turlova E., Wong R., Xu B., Li F., Du L., Habbous S., Horgen F.D., Fleig A., Feng Z.P., Sun H.S. (2021). TRPM7 Mediates Neuronal Cell Death Upstream. of Calcium/Calmodulin-Dependent Protein Kinase II and Calcineurin Mechanism in Neonatal Hypoxic-Ischemic Brain Injury. Transl. Stroke Res..

[36] Sarria I., Ling J., Zhu M.X., Gu J.G. (2011). TRPM8 acute desensitization is mediated by calmodulin and requires. PIP(2): Distinction from tachyphylaxis. J. Neurophysiol..

[37] Hof T., Chaigne S., Recalde A., Salle L., Brette F., Guinamard R. (2019). Transient receptor potential channels in cardiac health and disease. Nat. Rev. Cardiol..

[38] DeLano W.L. (2002). Pymol: An open-source molecular graphics tool. CCP4 Newsl. Protein Crystallogr..

[39] Latorre R., Díaz-Franulic I. (2022). Profile of David Julius and Ardem Patapoutian: 2021 nobel laureates in physiology or medicine. Proc. Natl. Acad. Sci. USA.

[40] Bagur R., Hajnoczky G. (2017). Intracellular Ca(2+) Sensing: Its Role in Calcium Homeostasis and Signaling. Mol. Cell.

[41] Sharma R.K., Parameswaran S. (2018). Calmodulin-binding proteins: A journey of 40 years. Cell Calcium.

[42] Halling D.B., Liebeskind B.J., Hall A.W., Aldrich R.W. (2016). Conserved properties of individual Ca^2+^-binding sites in calmodulin. Proc. Natl. Acad. Sci. USA.

[43] Jensen H.H., Brohus M., Nyegaard M., Overgaard M.T. (2018). Human Calmodulin Mutations. Front. Mol. Neurosci..

[44] Barbato G., Ikura M., Kay L.E., Pastor R.W., Bax A. (1992). Backbone dynamics of calmodulin studied by 15N relaxation using inverse detected two-dimensional NMR spectroscopy: The central helix is flexible. Biochemistry.

[45] Babu Y.S., Bugg C.E., Cook W.J. (1988). Structure of calmodulin refined at 2.2 A resolution. J. Mol. Biol..

[46] Grabarek Z. (2006). Structural basis for diversity of the EF-hand calcium-binding proteins. J. Mol. Biol..

[47] Liu X.R., Zhang M.M., Rempel D.L., Gross M.L. (2019). A Single Approach Reveals the Composite Conformational Changes, Order of Binding, and Affinities for Calcium Binding to Calmodulin. Anal. Chem..

[48] Lau S.Y., Procko E., Gaudet R. (2012). Distinct properties of Ca^2+^-calmodulin binding to N- and C-terminal regulatory regions of the TRPV1 channel. J. Gen. Physiol..

[49] Ataman Z.A., Gakhar L., Sorensen B.R., Hell J.W., Shea M.A. (2007). The NMDA receptor NR1 C1 region bound to calmodulin: Structural insights into functional differences between homologous domains. Structure.

[50] Johnson C.N., Potet F., Thompson M.K., Kroncke B.M., Glazer A.M., Voehler M.W., Knollmann B.C., George A.L., Chazin W.J. (2018). A Mechanism of Calmodulin Modulation of the Human Cardiac Sodium Channel. Structure.

[51] Liu Y., Zheng X., Mueller G.A., Sobhany M., DeRose E.F., Zhang Y., London R.E., Birnbaumer L. (2012). Crystal structure of calmodulin binding domain of orai1 in complex with Ca^2+^ calmodulin displays a unique binding mode. J. Biol. Chem..

[52] Strotmann R., Schultz G., Plant T.D. (2003). Ca^2+^-dependent potentiation of the nonselective cation channel TRPV4 is mediated by a C-terminal calmodulin binding site. J. Biol. Chem..

[53] Numazaki M., Tominaga T., Takeuchi K., Murayama N., Toyooka H., Tominaga M. (2003). Structural determinant of TRPV1 desensitization interacts with calmodulin. Proc. Natl. Acad. Sci. USA.

[54] Rosenbaum T., Gordon-Shaag A., Munari M., Gordon S.E. (2004). Ca^2+^/calmodulin modulates TRPV1 activation by capsaicin. J. Gen. Physiol..

[55] Xiao R., Tang J., Wang C., Colton C.K., Tian J., Zhu M.X. (2008). Calcium plays a central role in the sensitization of TRPV3 channel to repetitive stimulations. J. Biol. Chem..

[56] De Groot T., Kovalevskaya N.V., Verkaart S., Schilderink N., Felici M., van der Hagen E.A., Bindels R.J., Vuister G.W., Hoenderop J.G. (2011). Molecular mechanisms of calmodulin action on TRPV5 and modulation by parathyroid hormone. Mol. Cell. Biol..

[57] Derler I., Hofbauer M., Kahr H., Fritsch R., Muik M., Kepplinger K., Hack M.E., Moritz S., Schindl R., Groschner K. (2006). Dynamic but not constitutive association of calmodulin with rat TRPV6 channels enables fine tuning of Ca^2+^-dependent inactivation. J. Physiol..

[58] Hasan R., Leeson-Payne A.T., Jaggar J.H., Zhang X. (2017). Calmodulin is responsible for Ca(2+)-dependent regulation of TRPA1 Channels. Sci. Rep..

[59] Jung J., Shin J.S., Lee S.Y., Hwang S.W., Koo J., Cho H., Oh U. (2004). Phosphorylation of vanilloid receptor 1 by Ca^2+^/calmodulin-dependent kinase II regulates its vanilloid binding. J. Biol. Chem..

[60] Shi J., Mori E., Mori Y., Mori M., Li J., Ito Y., Inoue R. (2004). Multiple regulation by calcium of murine homologues of transient receptor potential proteins TRPC6 and TRPC7 expressed in HEK293 cells. J. Physiol..

[61] Hsieh C.-C., Su Y.-C., Jiang K.-Y., Ito T., Li T.-W., Kaku-Ito Y., Cheng S.-T., Chen L.-T., Hwang D.-Y., Shen C.-H. (2023). TRPM1 promotes tumor progression in acral melanoma by activating the Ca^2+^/CaMKIIδ/AKT pathway. J. Adv. Res..

[62] Wang Q., Guo W., Hao B., Shi X., Lu Y., Wong C.W., Ma V.W., Yip T.T., Au J.S., Hao Q. (2016). Mechanistic study of TRPM2-Ca(2+)-CAMK2-BECN1 signaling in oxidative stress-induced autophagy inhibition. Autophagy.

[63] Qian N., Ichimura A., Takei D., Sakaguchi R., Kitani A., Nagaoka R., Tomizawa M., Miyazaki Y., Miyachi H., Numata T. (2019). TRPM7 channels mediate spontaneous Ca(2+) fluctuations in growth plate chondrocytes that promote bone development. Sci. Signal..

[64] Bahler M., Rhoads A. (2002). Calmodulin signaling via the IQ motif. FEBS Lett..

[65] Rhoads A.R., Friedberg F. (1997). Sequence motifs for calmodulin recognition. FASEB J..

[66] Yap K.L., Kim J., Truong K., Sherman M., Yuan T., Ikura M. (2000). Calmodulin target database. J. Struct. Funct. Genom..

[67] Rodríguez-Castañeda F., Maestre-Martínez M., Coudevylle N., Dimova K., Junge H., Lipstein N., Lee D., Becker S., Brose N., Jahn O. (2010). Modular architecture of Munc13/calmodulin complexes: Dual regulation by Ca^2+^ and possible function in short-term synaptic plasticity. EMBO J..

[68] Osawa M., Tokumitsu H., Swindells M.B., Kurihara H., Orita M., Shibanuma T., Furuya T., Ikura M. (1999). A novel target recognition revealed by calmodulin in complex with Ca^2+^-calmodulin-dependent kinase kinase. Nat. Struct. Biol..

[69] Maximciuc A.A., Putkey J.A., Shamoo Y., Mackenzie K.R. (2006). Complex of calmodulin with a ryanodine receptor target reveals a novel, flexible binding mode. Structure.

[70] Juranic N., Atanasova E., Filoteo A.G., Macura S., Prendergast F.G., Penniston J.T., Strehler E.E. (2010). Calmodulin wraps around its binding domain in the plasma membrane Ca^2+^ pump anchored by a novel 18-1 motif. J. Biol. Chem..

[71] Yamauchi E., Nakatsu T., Matsubara M., Kato H., Taniguchi H. (2003). Crystal structure of a MARCKS peptide containing the calmodulin-binding domain in complex with Ca^2+^-calmodulin. Nat. Struct. Biol..

[72] Zouharova M., Herman P., Hofbauerova K., Vondrasek J., Bousova K. (2019). TRPM6 N-Terminal CaM- and S100A1-Binding Domains. Int. J. Mol. Sci..

[73] Holakovska B., Grycova L., Jirku M., Sulc M., Bumba L., Teisinger J. (2012). Calmodulin and S100A1 protein interact with N terminus of TRPM3 channel. J. Biol. Chem..

[74] Bousova K., Herman P., Vecer J., Bednarova L., Monincova L., Majer P., Vyklicky L., Vondrasek J., Teisinger J. (2018). Shared CaM- and S100A1-binding epitopes in the distal TRPM4 N terminus. FEBS J..

[75] Bousova K., Barvik I., Herman P., Hofbauerová K., Monincova L., Majer P., Zouharova M., Vetyskova V., Postulkova K., Vondrasek J. (2020). Mapping of CaM, S100A1 and PIP2-Binding Epitopes in the Intracellular N-and C-Termini of TRPM4. Int. J. Mol. Sci..

[76] Bousova K., Zouharova M., Herman P., Vymetal J., Vetyskova V., Jiraskova K., Vondrasek J. (2022). TRPM5 Channel Binds Calcium-Binding Proteins Calmodulin and S100A1. Biochemistry.

[77] Bousova K., Zouharova M., Herman P., Vetyskova V., Jiraskova K., Vondrasek J. (2021). TRPM7 N-terminal region forms complexes with calcium binding proteins CAm. and S100A1. Heliyon.

[78] Bily J., Grycova L., Holendova B., Jirku M., Janouskova H., Bousova K., Teisinger J. (2013). Characterization of the S100A1 protein binding site on TRPC6 C-terminus. PLoS ONE.

[79] Jirku M., Lansky Z., Bednarova L., Sulc M., Monincova L., Majer P., Vyklicky L., Vondrasek J., Teisinger J., Bousova K. (2016). The characterization of a novel S100A1 binding site in the N-terminus of TRPM1. Int. J. Biochem. Cell Biol..

[80] Palladino A., Papa A.A., Petillo R., Scutifero M., Morra S., Passamano L., Nigro V., Politano L. (2022). The role of TRPM4 gene mutations in causing familial progressive cardiac conduction disease: A further contribution. Genes.

[81] Wang H., Xu Z., Lee B.H., Vu S., Hu L., Lee M., Bu D., Cao X., Hwang S., Yang Y. (2019). Gain-of-function mutations in TRPM4 activation gate cause progressive symmetric erythrokeratodermia. J. Investig. Dermatol..

[82] Schlingmann K.P., Weber S., Peters M., Niemann Nejsum L., Vitzthum H., Klingel K., Kratz M., Haddad E., Ristoff E., Dinour D. (2002). Hypomagnesemia with secondary hypocalcemia is caused by mutations in TRPM6, a new member of the TRPM gene family. Nat. Genet..

[83] Lainez S., Schlingmann K.P., Van Der Wijst J., Dworniczak B., Van Zeeland F., Konrad M., Bindels R.J., Hoenderop J.G. (2014). New TRPM6 missense mutations linked to hypomagnesemia with secondary hypocalcemia. Eur. J. Hum. Genet..

[84] Leddy H.A., McNulty A.L., Guilak F., Liedtke W. (2014). Unraveling the mechanism by which TRPV4 mutations cause skeletal dysplasias. Rare Dis..

[85] Schlöndorff J., Del Camino D., Carrasquillo R., Lacey V., Pollak M.R. (2009). TRPC6 mutations associated with focal segmental glomerulosclerosis cause constitutive activation of NFAT-dependent transcription. Am. J. Physiol. Cell Physiol..

[86] Dang S., van Goor M.K., Asarnow D., Wang Y., Julius D., Cheng Y., van der Wijst J. (2019). Structural insight into TRPV5 channel function and modulation. Proc. Natl. Acad. Sci. USA.

[87] Hughes T.E., Pumroy R.A., Yazici A.T., Kasimova M.A., Fluck E.C., Huynh K.W., Samanta A., Molugu S.K., Zhou Z.H., Carnevale V. (2018). Structural insights on TRPV5 gating by endogenous modulators. Nat. Commun..

[88] Singh A.K., McGoldrick L.L., Twomey E.C., Sobolevsky A.I. (2018). Mechanism of calmodulin inactivation of the calcium-selective TRP channel TRPV6. Sci. Adv..

[89] Vinayagam D., Quentin D., Yu-Strzelczyk J., Sitsel O., Merino F., Stabrin M., Hofnagel O., Yu M., Ledeboer M.W., Nagel G. (2020). Structural basis of TRPC4 regulation by calmodulin and pharmacological agents. eLife.

[90] Venkatachalam K., Montell C. (2007). TRP channels. Annu. Rev. Biochem.

[91] Vriens J., Owsianik G., Hofmann T., Philipp S.E., Stab J., Chen X.D., Benoit M., Xue F.Q., Janssens A., Kerselaers S. (2011). TRPM3 Is a Nociceptor Channel Involved in the Detection of Noxious Heat. Neuron.

[92] Alonso-Carbajo L., Kecskes M., Jacobs G., Pironet A., Syam N., Talavera K., Vennekens R. (2017). Muscling in on TRP channels in vascular smooth muscle cells and cardiomyocytes. Cell Calcium.

[93] Thiel G., Rubil S., Lesch A., Guethlein L.A., Rossler O.G. (2017). Transient receptor potential TRPM3 channels: Pharmacology, signaling, and biological functions. Pharmacol. Res..

[94] Dyment D.A., Terhal P.A., Rustad C.F., Tveten K., Griffith C., Jayakar P., Shinawi M., Ellingwood S., Smith R., van Gassen K. (2019). De novo substitutions of TRPM3 cause intellectual disability and epilepsy. Eur. J. Hum. Genet..

[95] Wagner T.F., Loch S., Lambert S., Straub I., Mannebach S., Mathar I., Dufer M., Lis A., Flockerzi V., Philipp S.E. (2008). Transient receptor potential M3 channels are ionotropic steroid receptors in pancreatic beta cells. Nat. Cell Biol..

[96] Toth B.I., Konrad M., Ghosh D., Mohr F., Halaszovich C.R., Leitner M.G., Vriens J., Oberwinkler J., Voets T. (2015). Regulation of the transient receptor potential channel TRPM3 by phosphoinositides. J. Gen. Physiol..

[97] Badheka D., Borbiro I., Rohacs T. (2015). Transient receptor potential melastatin 3 is a phosphoinositide-dependent ion channel. J. Gen. Physiol..

[98] Holendova B., Grycova L., Jirku M., Teisinger J. (2012). PtdIns(4,5)P2 interacts with Cam. binding domains on TRPM3 N-terminus. Channels.

[99] Oberwinkler J., Philipp S.E. (2014). Trpm3. Handb. Exp. Pharmacol..

[100] Thiel G., Rössler O.G. (2023). Calmodulin Regulates Transient Receptor Potential TRPM3 and TRPM8-Induced Gene Transcription. Int. J. Mol. Sci..

[101] Prawitt D., Monteilh-Zoller M.K., Brixel L., Spangenberg C., Zabel B., Fleig A., Penner R. (2003). TRPM5 is a transient Ca^2+^-activated cation channel responding to rapid changes in [Ca^2+^]i. Proc. Natl. Acad. Sci. USA.

[102] Liu D., Liman E.R. (2003). Intracellular Ca^2+^ and the phospholipid PIP2 regulate the taste transduction ion channel TRPM5. Proc. Natl. Acad. Sci. USA.

[103] Zhang Z., Zhao Z., Margolskee R., Liman E. (2007). The transduction channel TRPM5 is gated by intracellular calcium in taste cells. J. Neurosci..

[104] Voets T., Nilius B., Hoefs S., van der Kemp A.W., Droogmans G., Bindels R.J., Hoenderop J.G. (2004). TRPM6 forms the Mg^2+^ influx channel involved in intestinal and renal Mg^2+^ absorption. J. Biol. Chem..

[105] Xie J., Sun B., Du J., Yang W., Chen H.C., Overton J.D., Runnels L.W., Yue L. (2011). Phosphatidylinositol 4,5-bisphosphate (PIP(2)) controls magnesium gatekeeper TRPM6 activity. Sci. Rep..

[106] Groenestege W.M., Hoenderop J.G., van den Heuvel L., Knoers N., Bindels R.J. (2006). The epithelial Mg^2+^ channel transient receptor potential melastatin 6 is regulated by dietary Mg^2+^ content and estrogens. J. Am. Soc. Nephrol..

[107] Runnels L.W., Yue L., Clapham D.E. (2002). The TRPM7 channel is inactivated by PIP(2) hydrolysis. Nat. Cell Biol..

[108] Nadler M.J., Hermosura M.C., Inabe K., Perraud A.L., Zhu Q., Stokes A.J., Kurosaki T., Kinet J.P., Penner R., Scharenberg A.M. (2001). LTRPC7 is a Mg·ATP-regulated divalent cation channel required for cell viability. Nature.

[109] Sisco N.J., Luu D.D., Kim M., Van Horn W.D. (2020). PIRT the TRP Channel Regulating Protein Binds Calmodulin and Cholesterol-Like Ligands. Biomolecules.

[110] Belrose J.C., Jackson M.F. (2018). TRPM2: A candidate therapeutic target for treating neurological diseases. Acta Pharmacol. Sin..

[111] Miller B.A., Wang J., Hirschler-Laszkiewicz I., Gao E., Song J., Zhang X.Q., Koch W.J., Madesh M., Mallilankaraman K., Gu T. (2013). The second member of transient receptor potential-melastatin channel family protects hearts from ischemia-reperfusion injury. Am. J. Physiol. Heart Circ. Physiol..

[112] Mittal M., Nepal S., Tsukasaki Y., Hecquet C.M., Soni D., Tiruppathi C., Malik A.B., Rehman J. (2017). Response by Mittal et al to Letter Regarding Article, “Neutrophil Activation of Endothelial Cell-Expressed TRPM2 Mediates Transendothelial Neutrophil Migration and Vascular Injury”. Circ. Res..

[113] Ito K., Dezaki K., Yoshida M., Yamada H., Miura R., Rita R.S., Ookawara S., Tabei K., Kawakami M., Hara K. (2017). Endogenous alpha2A-Adrenoceptor-Operated Sympathoadrenergic Tones Attenuate Insulin Secretion via cAMP/TRPM2 Signaling. Diabetes.

[114] Massullo P., Sumoza-Toledo A., Bhagat H., Partida-Sanchez S. (2006). TRPM channels, calcium and redox sensors during innate immune responses. Semin. Cell Dev. Biol..

[115] Yamamoto S., Shimizu S. (2016). Targeting TRPM2 in ROS-Coupled Diseases. Pharmaceuticals.

[116] McHugh D., Flemming R., Xu S.Z., Perraud A.L., Beech D.J. (2003). Critical intracellular Ca^2+^ dependence of transient receptor potential melastatin 2 (TRPM2) cation channel activation. J. Biol. Chem..

[117] Starkus J., Beck A., Fleig A., Penner R. (2007). Regulation of TRPM2 by extra- and intracellular calcium. J. Gen. Physiol..

[118] Tan C.H., McNaughton P.A. (2016). The TRPM2 ion channel is required for sensitivity to warmth. Nature.

[119] Song K., Wang H., Kamm G.B., Pohle J., Reis F.C., Heppenstall P., Wende H., Siemens J. (2016). The TRPM2 channel is a hypothalamic heat sensor that limits fever and can drive hypothermia. Science.

[120] Kashio M., Tominaga M. (2017). The TRPM2 channel: A thermo-sensitive metabolic sensor. Channels.

[121] Yu P., Xue X., Zhang J., Hu X., Wu Y., Jiang L.H., Jin H., Luo J., Zhang L., Liu Z. (2017). Identification of the ADPR binding pocket in the NUDT9 homology domain of TRPM2. J. Gen. Physiol..

[122] Togashi K., Hara Y., Tominaga T., Higashi T., Konishi Y., Mori Y., Tominaga M. (2006). TRPM2 activation by cyclic ADP-ribose at body temperature is involved in insulin secretion. EMBO J..

[123] Cai X., Yu X., Yang J., Lu L., Hua N., Duan X., Ye P., Ni L., Jiang L., Yang W. (2023). TRPM2 regulates cell cycle through the Ca^2+^-CaM-CaMKII signaling pathway to promote HCC. Hepatol. Commun..

[124] Harteneck C. (2005). Function and pharmacology of TRPM cation channels. Naunyn. Schmiedebergs Arch. Pharmacol..

[125] Fonfria E., Murdock P.R., Cusdin F.S., Benham C.D., Kelsell R.E., McNulty S. (2006). Tissue distribution profiles of the human TRPM cation channel family. J. Recept. Signal Transduct. Res..

[126] Xu X.-Z.S., Moebius F., Gill D.L., Montell C. (2001). Regulation of melastatin, a TRP-related protein, through interaction with a cytoplasmic isoform. Proc. Natl. Acad. Sci. USA.

[127] Launay P., Cheng H., Srivatsan S., Penner R., Fleig A., Kinet J.-P. (2004). TRPM4 regulates calcium oscillations after T cell activation. Science.

[128] Schattling B., Steinbach K., Thies E., Kruse M., Menigoz A., Ufer F., Flockerzi V., Bruck W., Pongs O., Vennekens R. (2012). TRPM4 cation channel mediates axonal and neuronal degeneration in experimental autoimmune encephalomyelitis and multiple sclerosis. Nat. Med..

[129] Borgstrom A., Peinelt C., Stoklosa P. (2021). TRPM4 in Cancer—A New Potential Drug Target. Biomolecules.

[130] Bianchi B., Ozhathil L.C., Medeiros-Domingo A., Gollob M.H., Abriel H. (2018). Four TRPM4 Cation Channel Mutations Found in Cardiac Conduction Diseases Lead to Altered Protein Stability. Front. Physiol..

[131] Daumy X., Amarouch M.Y., Lindenbaum P., Bonnaud S., Charpentier E., Bianchi B., Nafzger S., Baron E., Fouchard S., Thollet A. (2016). Targeted resequencing identifies TRPM4 as a major gene predisposing to progressive familial heart block type I. Int. J. Cardiol..

[132] Wang C., Naruse K., Takahashi K. (2018). Role of the TRPM4 Channel in Cardiovascular Physiology and Pathophysiology. Cells.

[133] Yamaguchi S., Tanimoto A., Otsuguro K., Hibino H., Ito S. (2014). Negatively charged amino acids near and in transient receptor potential (TRP) domain of TRPM4 channel are one determinant of its Ca^2+^ sensitivity. J. Biol. Chem..

[134] Nilius B., Prenen J., Janssens A., Voets T., Droogmans G. (2004). Decavanadate modulates gating of TRPM4 cation channels. J. Physiol..

[135] Nilius B., Mahieu F., Prenen J., Janssens A., Owsianik G., Vennekens R., Voets T. (2006). The Ca^2+^-activated cation channel TRPM4 is regulated by phosphatidylinositol 4,5-biphosphate. EMBO J..

[136] Zhang Z., Okawa H., Wang Y., Liman E.R. (2005). Phosphatidylinositol 4,5-bisphosphate rescues TRPM4 channels from desensitization. J. Biol. Chem..

[137] Hu Y., Kaschitza D.R., Essers M., Arullampalam P., Fujita T., Abriel H., Inoue R. (2021). Pathological activation of CaMKII induces arrhythmogenicity through TRPM4 overactivation. Pflug. Arch..

[138] Hofmann T., Chubanov V., Gudermann T., Montell C. (2003). TRPM5 is a voltage-modulated and Ca(2+)-activated monovalent selective cation channel. Curr. Biol..

[139] Dutta Banik D., Martin L.E., Freichel M., Torregrossa A.M., Medler K.F. (2018). TRPM4 and TRPM5 are both required for normal signaling in taste receptor cells. Proc. Natl. Acad. Sci. USA.

[140] Vennekens R., Mesuere M., Philippaert K. (2018). TRPM5 in the battle against diabetes and obesity. Acta Physiol..

[141] Maeda T., Suzuki A., Koga K., Miyamoto C., Maehata Y., Ozawa S., Hata R.-I., Nagashima Y., Nabeshima K., Miyazaki K. (2017). TRPM5 mediates acidic extracellular pH signaling and TRPM5 inhibition reduces spontaneous metastasis in mouse B16-BL6 melanoma cells. Oncotarget.

[142] Launay P., Fleig A., Perraud A.L., Scharenberg A.M., Penner R., Kinet J.P. (2002). TRPM4 is a Ca^2+^-activated nonselective cation channel mediating cell membrane depolarization. Cell.

[143] Ryazanova L.V., Rondon L.J., Zierler S., Hu Z., Galli J., Yamaguchi T.P., Mazur A., Fleig A., Ryazanov A.G. (2010). TRPM7 is essential for Mg(2+) homeostasis in mammals. Nat. Commun..

[144] Clark K., Middelbeek J., Morrice N.A., Figdor C.G., Lasonder E., van Leeuwen F.N. (2008). Massive autophosphorylation of the Ser/Thr-rich domain controls protein kinase activity of TRPM6 and TRPM7. PLoS ONE.

[145] Romagnani A., Vettore V., Rezzonico-Jost T., Hampe S., Rottoli E., Nadolni W., Perotti M., Meier M.A., Hermanns C., Geiger S. (2017). TRPM7 kinase activity is essential for T cell colonization and alloreactivity in the gut. Nat. Commun..

[146] Costa A., Tejpar S., Prenen H., Van Cutsem E. (2011). Hypomagnesaemia and targeted anti-epidermal growth factor receptor (EGFR) agents. Target. Oncol..

[147] Krapivinsky G., Krapivinsky L., Renthal N.E., Santa-Cruz A., Manasian Y., Clapham D.E. (2017). Histone phosphorylation by TRPM6’s cleaved kinase attenuates adjacent arginine methylation to regulate gene expression. Proc. Natl. Acad. Sci. USA.

[148] Zou Z.G., Rios F.J., Montezano A.C., Touyz R.M. (2019). TRPM7, Magnesium, and Signaling. Int. J. Mol. Sci..

[149] Abumaria N., Li W., Clarkson A.N. (2019). Role of the chanzyme TRPM7 in the nervous system in health and disease. Cell Mol. Life Sci..

[150] Miyazaki Y., Ichimura A., Kitayama R., Okamoto N., Yasue T., Liu F., Kawabe T., Nagatomo H., Ueda Y., Yamauchi I. (2022). C-type natriuretic peptide facilitates autonomic Ca^2+^ entry in growth plate chondrocytes for stimulating bone growth. eLife.

[151] Chubanov V., Ferioli S., Wisnowsky A., Simmons D.G., Leitzinger C., Einer C., Jonas W., Shymkiv Y., Bartsch H., Braun A. (2016). Epithelial magnesium transport by TRPM6 is essential for prenatal development and adult survival. eLife.

[152] McKemy D.D., Neuhausser W.M., Julius D. (2002). Identification of a cold receptor reveals a general role for TRP channels in thermosensation. Nature.

[153] Peier A.M., Moqrich A., Hergarden A.C., Reeve A.J., Andersson D.A., Story G.M., Earley T.J., Dragoni I., McIntyre P., Bevan S. (2002). A TRP channel that senses cold stimuli and menthol. Cell.

[154] Abe J., Hosokawa H., Okazawa M., Kandachi M., Sawada Y., Yamanaka K., Matsumura K., Kobayashi S. (2005). TRPM8 protein localization in trigeminal ganglion and taste papillae. Brain Res. Mol. Brain Res..

[155] Nealen M.L., Gold M.S., Thut P.D., Caterina M.J. (2003). TRPM8 mRNA is expressed in a subset of cold-responsive trigeminal neurons from rat. J. Neurophysiol..

[156] Winchester W.J., Gore K., Glatt S., Petit W., Gardiner J.C., Conlon K., Postlethwaite M., Saintot P.-P., Roberts S., Gosset J.R. (2014). Inhibition of TRPM8 channels reduces pain in the cold pressor test in humans. J. Pharmacol. Exp. Ther..

[157] Qin N., Flores C.M. (2007). Polypeptide Complex of TRPM8 and Calmodulin and Its Uses Thereof. U.S. Patent.

[158] Premkumar L.S., Raisinghani M., Pingle S.C., Long C., Pimentel F. (2005). Downregulation of transient receptor potential melastatin 8 by protein kinase C-mediated dephosphorylation. J. Neurosci..

[159] Iftinca M., Basso L., Flynn R., Kwok C., Roland C., Hassan A., Defaye M., Ramachandran R., Trang T., Altier C. (2020). Chronic morphine regulates TRPM8 channels via MOR-PKCβ signaling. Mol. Brain.

[160] Iftinca M., Altier C. (2020). The cool things to know about TRPM8!. Channels.

[161] Behrendt M. (2019). Transient receptor potential channels in the context of nociception and pain–recent insights into TRPM3 properties and function. Biol. Chem..

[162] Kruse M., Pongs O. (2014). TRPM4 channels in the cardiovascular system. Curr. Opin. Pharmacol..

